# The T-Cell Immune Response against Kaposi's Sarcoma-Associated Herpesvirus

**DOI:** 10.1155/2010/340356

**Published:** 2011-01-16

**Authors:** Rebecca C. Robey, Salvinia Mletzko, Frances M. Gotch

**Affiliations:** Department of Immunology, Imperial College London, Chelsea and Westminster Hospital, 369 Fulham Road, London SW10 9NH, UK

## Abstract

Kaposi's sarcoma-associated herpesvirus (KSHV) is the aetiological agent of Kaposi's sarcoma (KS), the most frequently arising malignancy in individuals with untreated HIV/AIDS. There are several lines of evidence to indicate that Kaposi's sarcoma oncogenesis is associated with loss of T-cell-mediated control of KSHV-infected cells. KSHV can establish life-long asymptomatic infection in immune-competent individuals. However, when T-cell immune control declines, for example, through AIDS or treatment with immunosuppressive drugs, both the prevalence of KSHV infection and the incidence of KS in KSHV carriers dramatically increase. Moreover, a dramatic and spontaneous improvement in KS is frequently seen when immunity is restored, for example, through antiretroviral therapy or the cessation of iatrogenic drugs. In this paper we describe the current state of knowledge on the T-cell immune responses against KSHV.

## 1. Introduction

Kaposi's sarcoma-associated herpesvirus (KSHV) (or human herpesvirus 8 (HHV8)) is an oncogenic herpesvirus that is the aetiological agent of Kaposi's sarcoma (KS), a mesenchymal tumour that is the most frequently arising cancer in individuals with untreated HIV/AIDs [1] and consequently one of the most common cancers in Sub-Saharan Africa [2]. KSHV is also involved in the pathogenesis of at least two lymphoproliferative disorders—primary effusion lymphoma (PEL) and multicentric Castleman's disease (MCD). 

In immunocompetent hosts, KSHV can establish persistent, asymptomatic infection. KSHV infection has been reported in 3 to 20 percent of US blood donors [3, 4] and 40 to 80 percent of the general population of East African countries [3, 5, 6]. However, upon immunosuppression (acquired or iatrogenic) both the seroprevalence of KSHV and the incidence of KS in KSHV carriers significantly increase. KS is more than 100 times more common in immunosuppressed individuals, such as HIV-infected individuals and transplant recipients, compared to immunocompetent individuals [1]. Individuals acquiring KSHV infection with preexisting HIV infection have a significantly higher risk of developing KS; almost 50% develop KS, indicating that in this setting KSHV is one of the most oncogenic human viruses known [7]. This indicates that loss of T-cell-mediated immune control allows KSHV-infected cells to proliferate unchecked and KSHV-related tumours to develop. Furthermore, a dramatic clinical improvement is seen in iatrogenic KS patients when general immunity is restored following the withdrawal of immunosuppressive drugs [8], and spontaneous tumour regression has been reported in individuals with AIDS-related KS (AIDS-KS) after starting anti-HIV therapy [9]. Recent evidence indicates that a positive clinical outcome is associated with the restoration of KSHV-specific immunity [10, 11]. Thus, successful immunity targeted against KSHV plays a key role in containing KSHV infection, enabling the virus to establish controlled life-long infection and to coexist with its host.

However, the relationship between the immune system and KS oncogenesis is extremely complex. An unusual feature of KS tumour biology is the important role played by inflammatory infiltrates, particularly at KS onset. Infiltration of the tumour site by peripheral blood mononuclear cells (PBMCs) that secrete high levels of Th1 cytokines precedes the formation of spindle (tumour) cells and appears to be essential to tumour formation [12]. Most KS lesions are polyclonal, and multiple lesions from the same individual are also mainly polyclonal [13, 14]. The accepted interpretation of these data is that KS starts as a hyperplastic polyclonal lesion that is associated with inflammation and KSHV infection that could give rise, under specific circumstances, to clonal metastatic lesions. This supports the idea that KS is only truly neoplastic in advanced stages [15]. KS thus exemplifies an inflammatory-driven oncogenic process, or paracrine neoplasia [16]. The development of KS may thus be seen as a multifactorial process in which: infection by KSHV is a requirement; immunosuppression is an important cofactor; and, paradoxically, some level of systemic and localised immune activation in the form of increased Th1 cytokine production (induced either by KSHV infection, HIV infection, or unknown causes) is also involved.

## 2. The Targets of the KSHV-Specific T-Cell Response

Identifying the targets of the host's cellular immune responses is important to our understanding of how KSHV infections are controlled in immunocompetent individuals and is a crucial step towards developing treatments such as immunotherapies, or even vaccines, against KSHV-related diseases. 

T-cell responses to KSHV have been studied mostly in KS patients and asymptomatic carriers of KSHV. Responses have been detected against several lytic and latent proteins [17–28, 31, 34, 38]. Some of these responses have been demonstrated to be functionally cytotoxic *in vitro *[17, 18], and there is some evidence they exert evolutionary pressure on the virus *in vivo* [19]. A few HLA-restricted KSHV-specific T-cell epitopes have been identified (see [19–28] summarised in Table 1). However, these are almost exclusively CD8 epitopes, and they elicit weak T-cell responses compared to epitopes from other viruses, such as HIV-1 and Epstein-Barr virus ((EBV), a human *γ*-herpesvirus closely related to KSHV) [20, 21]. Neither the breadth of the antigenic repertoire of the KSHV-specific T-cell immune response, nor its immunodominant targets, are fully understood. 

Our recent studies indicated that both the CD8 and CD4 T-cell responses against KSHV preferentially target early and late lytic gene products [31]. This appears to contrast with what is observed in the CD8 response against EBV, which is heavily skewed towards EBV genes expressed in the immediate-early stage of the lytic cycle [32]. However, it is in keeping with recent observations in the CD8 response against the murine *γ*-herpesvirus MHV68 (more closely related to KSHV than to EBV), which also preferentially targets early and late lytic gene products [33].

## 3. The CD8 T-Cell Response against KSHV

### 3.1. CD8 T-Cell Responses to Primary Infection

Unlike primary EBV infection, which in adolescents and adults often results in acute infectious mononucleosis, primary KSHV infection appears not to be associated with any specific severe illness. Studies of cohorts at risk of KSHV infection have identified adults and children undergoing KSHV seroconversion and report occasional mild symptoms of fatigue, diarrhoea, fever, and localised rash [34, 35]. The lack of notable symptoms makes identifying primary KSHV infection a challenge, and thus little is known about the T-cell response to KSHV at acquisition. A 15-year longitudinal study of HIV-negative adults at risk of KSHV infection identified five individuals who had seroconverted during the study period and retrospectively analysed cryopreserved PBMCs from blood samples taken from these individuals for several years before and after KSHV seroconversion [34]. The authors first looked for global changes in T-cell phenotype around the time of KSHV seroconversion, as expansion of activated CD8 T cells is a classic finding in EBV mononucleosis in adults [36, 37]. They observed no generalised expansion of CD8 or CD4 T-cell populations after primary KSHV infection, and no changes in the expression of naïve and memory markers CD45RA and CD45RO, or of activation and costimulatory markers CD38, CD28, and HLA-DR [34]. The authors were, however, able to detect KSHV-specific CD8 T-cell responses directed against a broad spectrum of immediate-early, early, and late lytic KSHV ORFs. Such responses were observed at the time of primary KSHV infection in all KSHV seroconverters. The authors observed no dominant CTL target between the study subjects [34].

A second study examined two HLA-A2-positive KSHV-negative recipients of kidneys from KSHV-positive donors [23]. Both recipients remained consistently clear of detectable KSHV infection for 24 and 11 months after transplantation, respectively. Strikingly, CD8 T cells specific for both lytic and latent KSHV ORFs were detected in one of these recipients whose donor was also HLA-A2-positive. The second recipient, whose donor was HLA-A2-negative, showed no detectable response. The authors, therefore, suggest that KSHV-specific CTLs are restricted by shared donor/recipient HLA alleles, in this case HLA-A2. In the first recipient, the majority of tetramer-positive CD8 T cells were of a terminally differentiated effector memory phenotype (CD45RA+CCR7−) and expressed perforin, indicating that the generation of a functional KSHV-specific CTL response can lead to abortive infection [23]. In keeping with this, KSHV-specific T-cell responses have also been detected in KSHV-seronegative individuals defined as being at high risk of KSHV infection [38]. The authors argued that overall evidence from this study indicated that those individuals who did not show a detectable KSHV serologic response, but who showed positive KSHV-specific T-cell responses, were indeed KSHV-infected. It is possible, therefore, that a very low level of viral replication may be sufficient to prime a KSHV-specific T-cell immune response that may confer protection against chronic KSHV infection. However, it is perhaps more likely that the current serological and PCR-based methods for detecting KSHV infection are not sensitive enough to identify low-level latent infection.

### 3.2. Frequency and Diversity of CD8 T-Cell Responses in Asymptomatic Infection and Disease

In the study of five KSHV seroconverters discussed above, the frequencies of both CD8 CTL precursors and CD8 IFN*γ*-producing cells, directed against lytic KSHV antigens, increased to a peak one-to-two years after primary infection, after which they decreased in correlation with declines in antibody titres, possibly due to decreased viral replication and lower antigenic burden [34]. One study reported that T-cell responses to KSHV increased with viral load in the peripheral blood and were more readily detectable in individuals with active KS than those who did not present with active KS [38]. However, other groups have been unable to confirm this, and there is otherwise strong evidence (discussed below) that high levels of KSHV-specific CD8 T-cell responses confer protection against KS oncogenesis. Responses to KSHV CD8 peptides have been found to be of higher frequency and of greater diversity in their antigenic repertoire in asymptomatic carriers of KSHV compared to those with either AIDS-related, classic, or iatrogenic KS [22, 23, 29]. KSHV-specific T-cell responses appeared concurrent with clinical improvement in iatrogenic KS patients after a reduction of their immunosuppressive therapy or a conversion from calcineurin inhibitors (which block calcineurin-activated transcription of IL2) to sirolimus (also known as rapamycin, which acts through mTOR to inhibit responses to IL2) [29]. A longitudinal study of an iatrogenic KS patient who presented with recurrent episodes of remission and relapse of KS lesions found a correlation between reduced frequency of KSHV-specific CD8 T cells and recurrence of active KS [23]. Furthermore, both the magnitude and the frequency of responses to KSHV CD8 peptides increase with immune reconstitution through HAART, which apparently correlates with spontaneous KS regression [11, 28].

To address whether low frequencies of KSHV-specific CD8 T cells in the peripheral blood of KS patients is due to recruitment of these cells to the site of the tumour, one group performed *in situ* tetramer staining, and confocal laser scanning microscopy on KS biopsy specimens from two patients who had detectable circulating KSHV-specific CD8 T cells at the time of biopsy. They found large numbers of KSHV-tetramer-negative CD8 T-cell infiltrates in the vicinity of KSHV LANA1-positive spindle (tumour) cells, but observed very few CD8 T cells that costained with KSHV tetramers. The few tetramer-positive CD8 infiltrates that were seen were mainly found in LANA1-negative tissue [23]. Thus, in this study, KSHV-tetramer-specific CD8 T cells did not appear to be preferentially recruited to inflamed tumour tissue. Further investigation is warranted to confirm these findings and to understand their biological relevance.

Together, the above data indicate that KS oncogenesis is associated with loss of CD8 T cell-mediated control of KSHV-infected cells. Interestingly, a study investigating KSHV-specific CD8 T-cell responses in multicentric Castleman's disease (MCD) found that individuals with MCD had similar frequencies of KSHV-specific CD8 T-cell responses, and these were directed against a similar antigenic repertoire, as compared to asymptomatic KSHV carriers [30]. Another group also reported high numbers of IFN*γ*-secreting KSHV-specific CD8 T-cells in two individuals with MCD [39]. This is in direct contrast to what is observed in KS and indicates that whilst KSHV-specific CD8 T cells may confer protection against the emergence of KS, they do not apparently protect against the development of MCD.

### 3.3. Functional Properties and Phenotypes of KSHV-Specific CD8 T Cells in Asymptomatic Infection and Disease

The precise role of KSHV-specific CD8 T cells in the pathogenesis or control of KSHV-related diseases may additionally depend on the functional properties and differentiation phenotypes of these cells. EBV-specific and HIV-specific CD8 T cells have been shown to produce a range of cytokines besides interferon IFN*γ* [40], and there is evidence that polyfunctional T-cell responses may be a correlate of control of HIV-infection [41]. However, little is known about the functionality of KSHV-specific CD8 T cells in asymptomatic infection and disease. Both monofunctional and polyfunctional responses to a number of epitopes were reported in HIV-negative asymptomatic carriers of KSHV [24]. One study compared cytokine release by CD8 T cells from four individuals with AIDS-KS that spontaneously regressed after initiation of HAART (“KS nonprogressors”) with cytokine release by CD8 T cells from three individuals with AIDS-KS that progressed and required additional chemotherapy, despite initiation of HAART (“KS progressors”) [42]. It was shown that KSHV-specific CTL responses from KS nonprogressors were more frequently polyfunctional (production of both IFN*γ* and TNF*α*) than CTL responses from KS progressors. In contrast, KSHV-specific CD8 T cells from both individuals with MCD and asymptomatic carriers of KSHV were demonstrated to be polyfunctional (secretion of two or more of IFN*γ*, IL2, TNF*α*, MIP1ß, or mobilisation of CD107a) after stimulation with pools of both lytic and latent peptides [30]. A study comparing the functionality of KSHV-specific CD8 T cells directed against lytic or latent antigens in HIV-positive asymptomatic carriers of KSHV found that multifunctional KSHV-specific T cells that responded to latent antigens were more frequently detected in CD8 T-cell populations than those targeted to lytic antigens, in keeping with observations in EBV-specific T cells [20]. 

Human memory CD8 and CD4 T cells can be divided into phenotypic subsets based on their functions and expression of certain cell surface markers. These subsets are broadly defined as central memory (T_CM_), effector memory (T_EM_), and terminally differentiated effector memory (T_EMRA_: a subset of CD8 T cells only) [43]. Effector memory T cells can be further subdivided into early-, intermediate-, and late-effector memory cells [44]. T_CM_ cells have little or no effector function but migrate to lymph nodes and readily proliferate and differentiate into T_EM_ cells in response to stimulation by their specific antigen [45]. T_EM_ cells migrate to inflamed tissues and are characterised by rapid effector function upon antigenic stimulation. Early and intermediate T_EM_ cells have low cytolytic activity and maintain high proliferative capacity. Late T_EM_ cells have less proliferative capacity but strong effector cytolytic function [44, 46]. T_EMRA_ cells are a unique subclass of CD8 T_EM_ cells that are fully differentiated, strongly cytolytic, carry large amounts of perforin, and are nonreplicative. They are a hallmark of a prolonged immune response [43]. The expression of several cell-surface molecules can be used to define memory subsets. 

In a study of three iatrogenic KS patients, conversion of their immunosuppressive drug regime from calcineurin inhibitors to sirolimus (also known as rapamycin, as described above) led to an increase in the frequency of naïve and central memory T cells in the general population of circulating CD8 T cells in conjunction with KS regression [29]. A recent study found, unexpectedly, that treating mice with rapamycin (previously classified as an immunosuppressive drug, as it inhibits responses to IL2) following infection with lymphocytic choriomeningitis virus (LCMV) enhanced both the quality and quantity of central memory virus-specific T cells [47]. Thus, converting these iatrogenic KS patients to sirolimus may be acting to promote an enrichment of their central memory CD8 T cell pool, and these cells may be playing an important role in keeping KSHV-infected cells in check and promoting regression of KS lesions.

There are few studies of the differentiation phenotype of KSHV-specific CD8 T cells. The few reports to date are somewhat contradictory and on the whole rather limited, particularly in terms of sample numbers. This, coupled with the complication of the background of immunosuppression (acquired or iatrogenic) in which the studies are conducted, means that a definitive understanding of the differentiation phenotype of KSHV-specific T cells remains elusive. 

Six transplant recipients who spontaneously controlled KSHV infection had significantly higher proportions of CD45RA+CCR7− T_EMRA_ KSHV-specific cells and significantly lower proportions of CD45RA−CCR7− T_EM_ KSHV-specific cells, compared to seven patients with active KS and five patients with KS in remission [23]. In six MCD patients, the majority of KSHV-specific CD8 T cells were also CD45RA−CCR7− T_EM_ cells. However, in this study, the dominance of this phenotype did not differ from that of seven HIV-positive asymptomatic carriers of KSHV. Furthermore, in the MCD patients, a significantly higher proportion of KSHV-specific CD8 T cells were found to be CD45RA−CCR7−CD27− late effector memory T cells and a correspondingly lower proportion were CD45RA−CCR7−CD27+ early and intermediate effector memory T cells, as compared to asymptomatic carriers [30]. This more differentiated phenotype correlated with increased viral load, and was postulated to result from high antigenic burden.

Two studies have compared the phenotypes of KSHV-specific T cells targeting either lytic or latent antigens. One group reportedly observed no difference between the differentiation phenotype of KSHV-specific CD8 T cells targeted against latent or lytic antigens [23]. However, it was not made clear whether or not they compared T cells specific for different antigens from within the same individual, or if they only compared lytic-antigen-specific T cells from one individual with latent-antigen-specific T cells from another individual. Neither was it clear how many responses were investigated, nor in how many individuals. Another group reported that CD8 T cells that recognised latent KSHV antigens were predominately of a CD45RA−CCR7− T_EM_ phenotype, with a low proportion of cells of a CD45RA+CCR7− T_EMRA_ phenotype whereas a much higher proportion of CD8 T cells directed against lytic KSHV antigens were CD45RA+CCR7− T_EMRA_ [20]. This is in keeping with observations of the memory phenotypes of EBV-specific T cells [48]. Interestingly, KSHV-specific T cells were overall more differentiated (higher proportions of CD45RA+CCR7− cells) than EBV-specific T cells [20].

## 4. The CD4 T-Cell Response against KSHV

The CD4 T-cell response against KSHV remains largely unexplored. Although some studies have looked at responses to KSHV by mixed CD8 and CD4 T-cell populations [10, 20, 38], there have been very few investigations specifically into the CD4 T-cell response against KSHV. One of the studies with mixed T cells reported that two samples out of 11 tested showed borderline CD4 T-cell reactivity [20]. They did not state which of the two antigens they were testing (ORF57 (lytic) and ORF73 (latent)) initiated these CD4 responses. Another group reported the identification of two CD4 T-cell epitopes (the only ones described to date) from within the latent antigens K12 and K15 in one individual with AIDS-KS [22]. In a few individuals, our group was able to detect CD4 responses to monocyte-derived dendritic cells lentivirally transduced to express KSHV antigens [31]. These were less frequently detected than CD8 responses but appeared to preferentially target early and late lytic antigens. The longitudinal study of three iatrogenic KS patients described in the CD8 response section above reported the emergence of CD4 responses to K12 (latent) and K8.1 (late lytic), in conjunction with KS regression in two of these three individuals [29]. The single individual in whom no KSHV-specific CD4 responses were observed was the only one out of the three that did not achieve full remission of their KS. The authors suggested that this was indicative of the importance of KSHV-specific CD4 responses in controlling KSHV infection. Although the small sample number and limited number of antigens make it difficult to reach a firm conclusion from this study, it seems likely that CD4 T cells may play a key role in the immune response against KSHV. 

The final phases of KSHV virion assembly occur in the endosomal cellular compartments with extensive targeting of viral proteins to endosomes. Thus, viral proteins can be efficiently processed through the intracellular endosome pathway, resulting in the presentation of CD4-specific viral epitopes through MHC-II to helper T cells. Processing of the EBV antigen EBNA1 for presentation in the context of MHC-II is also known to occur through the autophagy pathway [49]. Furthermore, the presentation of the EBV antigens EBNA2, EBNA3C and BHRF1 through MHC-II occurs by intercellular transfer of an antigenic moiety [50–52]. This process does not require cell contact, and the antigenic particle is taken up by neighbouring cells and processed as exogenous antigen for MHC-II-mediated presentation. It seems reasonable that one or all of these pathways may also be used for the presentation of KSHV antigens through MHC-II. 

The lack of known CD4 epitopes or antigens has limited studies into the association between KSHV-specific CD4 responses and the control of KSHV or the development (and subsequent resolution) of KSHV-related neoplasms. Low CD4 counts in persons infected with HIV are associated with the incidence of KS, and KS can spontaneously regress with immune reconstitution through HAART. A weak association was reported between increased CD4 counts after starting HAART and reconstitution of KSHV-specific immune responses [10]. Interestingly, however, clinical improvement of KS after initiation of HAART was not found to be associated with increased CD4 count, although it was significantly related to decreased HIV viral load [53]. 

The absence of known targets of the KSHV-specific CD4 response has also restricted the investigation of the functionality (Th1 versus Th2) and the differentiation phenotypes of KSHV-specific CD4 T cells in chronic infection and disease. In the three iatrogenic KS patients mentioned above, complete KS regression in two of the patients was associated with an expansion of the naïve and central memory compartments of the total circulating CD4 T-cell population. In the third patient, who did not achieve complete resolution of their KS, there was no enrichment of their central memory CD4 T-cell compartment, consistent with these cells playing a role in KS control. 

## 5. ***γδ*** T Cells in the Control of KSHV

CD4 and CD8 T cells make up the majority of CD3 T cells found in the body and are both characterised by T-cell receptors comprised of an *α*-chain and a *β*-chain. A small proportion of CD3 T cells have T-cell receptors made up of a *γ*-chain and a *δ*-chain and are thus known as *γδ* T cells. *γδ* T cells typically account for less than five percent of circulating T cells, but are enriched in epithelial-rich tissues such as the skin and intestines [54]. There are two main subtypes of *γδ* T cells, designated V*δ*1 and V*δ*2. In certain disease states, the representation of V*δ*1 and V*δ*2 shifts dramatically, for example in HIV-1 infection, V*δ*2 cells are lost and V*δ*1 cells expand [55, 56]. Although the significance of such changes is not understood, they imply a role for *γδ* T cells in antiviral immune responses [54].

One group has examined the involvement of *γδ* T cells in the control of chronic KSHV infection [57]. They observed a significant expansion of *γδ* T cells of the V*δ*1 subtype in the peripheral blood of HIV-negative asymptomatic carriers of KSHV, as compared to age-matched, HIV-negative, and KSHV-negative healthy controls. V*δ*1 T-cell expansion has been previously described in two instances: in all stages of HIV infection [55, 56] and in transplant recipients with active CMV infection [58]. Asymptomatic KSHV infection is, therefore, the second viral infection (after HIV) in which specific, long-lasting V*δ*1 T-cell expansion is observed during chronic stages of infection, and the only viral infection in which V*δ*1 expansion has been documented in immunocompetent individuals. Barcy and colleagues [57] further found that in asymptomatic carriers of KSHV, the *γδ* V*δ*1 T-cell subpopulation displayed an increase in the relative frequency of cells expressing an effector phenotype compared to KSHV-negative controls. *In vitro* experiments demonstrated V*δ*1 T-cell activation in response to infectious KSHV particles; KSHV-infected cell lines; and the KSHV viral proteins glycoprotein B (encoded by ORF8), K8.1 and ORF65 [57]. Moreover, V*δ*1 T cells prevented the release of infectious KSHV virions from KSHV-infected cell lines following the induction of lytic replication [57].

## 6. Future Perspective

There is still much to learn about the adaptive T-cell responses against KSHV, and the evidence examined above highlights the difficulty in detecting these weak responses as a major obstacle in the field, both in the work completed to date and for future investigations. Although some CD8 epitopes have been identified, it seems reasonable that there may be immunodominant epitopes yet to be determined. It is evident that the targets of the KSHV-specific CD4 response remain poorly understood. Further characterisation of the functionality and differentiation phenotypes of both CD8 and CD4 KSHV-specific T cells will be greatly aided by first achieving a better understanding of the targets of these cells. Such future investigations may assist the design of targeted therapeutic strategies to restore KSHV-specific T cell function, thus controlling KSHV infection in both AIDS and transplant recipients.

## Figures and Tables

**Table 1:** Known immunogenic KSHV open reading frames (ORFs) and CD4 and CD8 epitopes.

T-cell subtype	Expression	KSHV ORF	Epitope position	Epitope sequence	HLA restriction	Other responsive HLA types	Reference
Not determined	Latent(?) and lytic	K10.5	Not determined	Not determined	N/A	N/A	[20]

CD4	Latent and induced in lytic cycle	K12	aa46–60	RGPVAFRTRVATGAH	Not determined	N/A	[22]
		K15	aa171–185	GNIKLVSSVSFICAG	Not determined	N/A	[22]

CD4	Late lytic	K8.1	Not determined	Not determined	N/A	N/A	[29]

CD8	Latent	ORF 73	aa71–79	FTSGLPAFV	A2	A26/A29	[22]
			aa140–148	PESSQRPPL	A*0201	None identified	[24]
			aa238–246	WATESPIYV	A2	A26/A29; A39/A69	[22]
			aa281–289	AMLVLLAEI	A*0201	None identified	[24]
			aa417–425	DGGDGNKTL	A*0201	None identified	[24]
			aa688–697	QQDEQQQQDE	A*0201	None identified	[24]
			aa920–928	PVVSTHEQI	A*0201	None identified	[24]
			aa1116–1124	QMARLAWEA	A2	A26/A29; A2/A69	[22]

CD8	Latent and induced in lytic cycle	K12	aa17–25	LLNGWRWRL	A*0201	None identified	[21]
			aa16–25	VLLNGWRWRL	A2	None identified	[25]
			aa23–32	WRLGAIPPLV	A*0201	None identified	[24]
			aa31–45	LVCLLAISVVPPSGQ	B7	B7/B53; B7/B14;	[22]
						B35/B49; B7/B51	
		K15	aa123–131	ILFTSTFAV	A2	A2/A24	[22]
			aa155–163	FTLSLPFLY	B44	B44/B49	[22]
			aa166–175	ATVKTGNIKL	B7	None identified	[30]

CD8	Immediate-early lytic	K5	aa154–163	ALYAANNTRV	A2	None identified	[26]
		ORF 6	aa1050–1058	VLGDEVLSL	A*0201	None identified	[23]
		ORF 57	aa212–220	ISARGQELF	B57	B58?	[20]

CD8	Early lytic	K3	aa72–81	LPRLTYQEGL	B7	None identified	[26]
			aa96–105	GLAAATWVWL	A2	None identified	[26]
			aa127–137	FVFYQLFVV	A2	None identified	[26]
		ORF 8	aa159–168	SSMKVNVNGV	A*0201	None identified	[24]
			aa492–500	LMWYELSKI	A2	None identified	[27]
			aa736–745	MLMIIIVIAI	A*0201	None identified	[24]
		ORF 61	aa505–513	GLADVFAEL	A*0201	None identified	[23]
		ORF 65	aa35–43	NMSQAEYLV	A*0201	None identified	[23]
		ORF 70	aa108–116	VVQELLWFL	A2	None identified	[26]
			aa255–264	SLLTYMLAHV	A2	None identified	[26]
			aa259–267	YMLAHVTGL	A2	None identified	[26]
			aa236–245	LYQRSGDMGL	A24	None identified	[26]
			aa253–261	SYSLLTYML	A24	None identified	[26]
			aa296–305	TPRPFPRLEI	B7	None identified	[26]
CD8	Late lytic	K1	aa58–66	FRLTERTLF	Cw3	None identified	[19]
			aa82–90	HRQSIWITW	B*2702	None identified	[19]
			aa91–99	YPQPVLQTL	B51	None identified	[19]
			aa93–101	QPVLQTLCA	B55	None identified	[19]
		K8.1	aa73–81	RLAAGSPSS	A*0201	None identified	[24]
			aa131–145	ELTDALISAFSGSYS	A24	None identified	[28]
			aa135–143	ALISAFSGS	A*0201	None identified	[24]
			aa211–225	LILYLCVPRCRRKKP	Not determined	N/A	[28]
			aa209–217	LVLILYLCV	A2	None identified	[11]
		ORF 22	aa59–68	FLNWQNLLNV	A2	None identified	[25]
		ORF 25	Not determined	Not determined	N/A	N/A	[18]
		ORF 26	aa103–111	FQWDSNTQL	A2	None identified	[26]
			aa125–134	IVLESNGFDL	A2	None identified	[26]
			aa203–211	VLDDLSMYL	A2	None identified	[26]
